# Roadway roof shear slip movement mechanism induced by the strong dynamic pressure while ultra-thick coal seam mining

**DOI:** 10.1038/s41598-026-46440-x

**Published:** 2026-05-28

**Authors:** Chao Liu, Fangtian Wang, Yujun Zuo, Chen Wang, Chao Gao, Yan Lu, Wenjibin Sun, Wenhua Hao, Tiankuo Tang, Yang Zhang

**Affiliations:** 1 School of Mines, China University of Mining and Technology, Xuzhou, 221116 China; 2 State Key Laboratory for Fine Exploration and Intelligent Development of Coal Resources, China University of Mining and Technology, Xuzhou, 221116 China; 3 Mining College, Guizhou University, Guiyang, 550025 China; 4 Shanxi Ningwu Yushupo Coal Industry Co., Ltd., Xinzhou, 036702 Shanxi China; 5 Chengjiao Coal Mine, Henan Zhenglong Coal Industry Co., Ltd., Yongcheng, 476600 China

**Keywords:** Ultra-thick coal seams, Fully-mechanized top-coal caving mining, High-stress concentrations, Shear slip movement, Roof collapse, Coordinated control, Energy science and technology, Engineering

## Abstract

To clarify the roof shear slip movement mechanism of roadways subjected to strong dynamic pressure during ultrathick coal seam mining, this study investigates the return airways of the 5107 fully mechanized top-coal caving (FMTC) panel at Yushupo Mine as a case study. The deformation characteristics and structural instability of surrounding rock in high-stress roadways were systematically analyzed through field observations, theoretical modeling, numerical simulation, and engineering verification. Based on the limit equilibrium theory, a dynamic pressure thick coal seam roadway roof shear slip mechanics model was established, and the spatial distribution of the shear slip zone and the minimum effective anchorage thickness were quantitatively determined. A new integrated collaborative control strategy of “bending resistance shear control displacement” was proposed for ultra thick coal seam roadway, and on-site practice was carried out. The results indicate that the roof shear slip surface initiates approximately 0.53 m from the roadway rib, propagates toward the centerline, and reaches its maximum development at a height of 4.58 m, located only 0.06 m from the centerline. The pronounced reduction in safety factors near the centerline reveals a critical zone highly susceptible to roof instability induced by strong mining-induced dynamic pressure. Discrete element method (DEM) simulations were employed to quantify the effects of key controlling parameters, including roof coal cohesion, parting layer position, and support configurations, on the evolution of roof shear slip movement. On this basis, a coordinated control strategy integrating bending resistance, shear strengthening, and displacement control was proposed. A full-length anchored cable reinforcement scheme tailored for ultrathick coal seams was developed and implemented in the field. Monitoring results demonstrate that the proposed support system effectively controls surrounding rock deformation, optimizes bolt–cable load transfer, suppresses bed separation, and significantly enhances roof stability. Field application confirms its effectiveness in mitigating large deformation in surrounding rock and ensuring the safe and efficient mining of ultrathick coal seams.

## Introduction

**Fig. 1:**
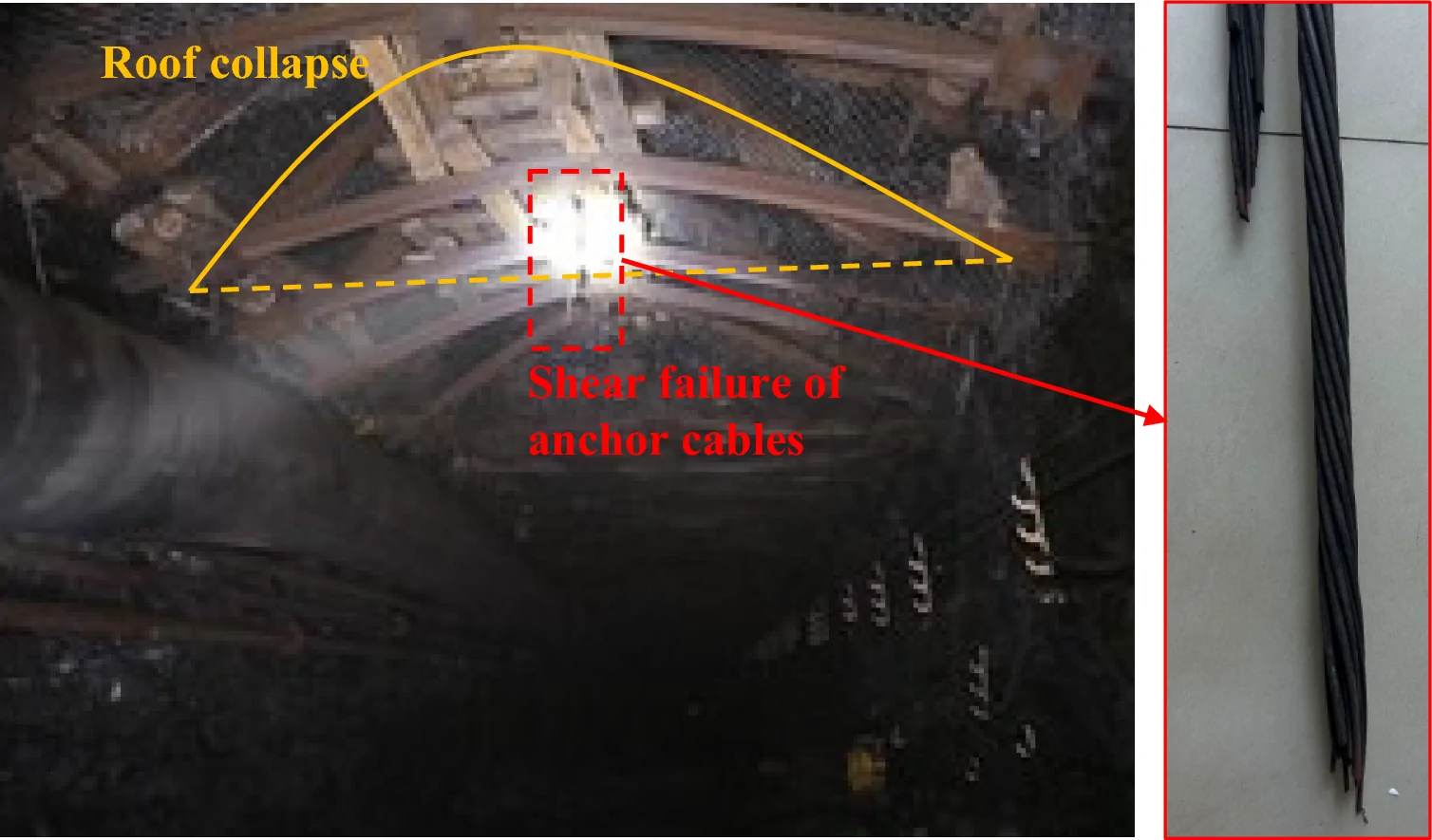
Roadway roof shear slip movement.

Advances in coal mining technology and equipment have enabled greater extraction heights and intensities, intensifying stability challenges in roadways within high-stress zones of ultrathick coal seams during fully mechanized top-coal caving (FMTC)^1–3^. In such roadways, roof coal serves dual functions: as the extraction target for FMTC operations and as a critical structural component of the roadway roof^4^. When subjected to intense lateral abutment pressure from adjacent gobs and mining-induced disturbances, roof coal experiences severe stress redistribution, frequently triggering instability or collapse^5,6^, threatens the safe operation of roadway equipment and personnel passages (Fig. 1), and severely constrains safe and efficient resource recovery.

Researchers worldwide have extensively studied the mechanisms of compound roof collapse in roadways through theoretical and experimental approaches. Dou et al.^7^ identified high static stress, dynamic disturbances, and inadequate support resistance as primary triggers. Roadways with thick roof coal exhibit greater roof deformation under dynamic loading than those with thin or noncoal roofs do, indicating heightened sensitivity to such disturbances. Chen et al.^8,9^ revealed the deformation and failure mechanism of the surrounding rock in a roadway and reported that the overall impact of a dynamic disturbance on the surrounding rock first intensifies and then weakens. Xu et al.^10,11^ established that roof failure depends on strength, thickness, stratigraphic position, overburden loading, and critical gob span. Under dynamic loading from superimposed thick-hard roof strata failure, the collapse risk increases significantly. Wang et al.^12^ identified transverse loading, effective roadway span, and coal‒rock strength as primary damage controls. High-stress conditions promote tensile‒shear failure in roof strata, where increasing the cable bolt density and pretension enhances the initial integrity. Yiouta-Mitra and Sofianos^13^ introduced a fresh perspective using catenary arch theory to analyze the buckling, compression, and slip mechanisms in jointed roof strata under gravitational loading. Ji et al.^14^ demonstrated that fracture depth governs roof failure mechanics, with crack position and extent critically influencing stress distribution.

Liu et al.^15^ developed segmented control technology that combines large-diameter stress-relief drilling with targeted cable reinforcement to mitigate mining-induced roadway deformation. Liu et al.^16,17^ proposed a stability control framework featuring stress field modification, structural reinforcement optimization, and critical zone support for roadways under compound mining effects. Fan et al.^18^ revealed that multiaxial stress fields cause conventional cable bolts to fracture and deform, prompting their design of energy-absorbing cables that effectively control surrounding rock failure. Xu et al.^10,11^ innovatively identified the sliding displacement of the main sliding zone as the key control objective. By suppressing the slip of the main slip zone, the formation of a multilayer compressive arch is promoted, and thereby, the stability of the comprehensive surrounding rock can be achieved. Wang et al.^19^ examined failure mechanisms in conventional gob-side entries and developed a high-strength bolting system incorporating surrounding rock mechanical parameters. Wang et al.^20^ investigated deformation mechanisms under mining disturbances and identified shear‒sliding mechanics in pillar ribs, which informed their inclined cable support design for roadway ribs.

While existing studies provide thorough analyses of roof instability mechanisms^8,9,21^, research on shear slip movement mechanics and stability control in roof coal under high-stress conditions within ultrathick seams is limited. Crucially, controlling shear-induced coal blocks directly impacts shear slip movement prevention, which is a pivotal factor for roadway stability in ultrathick seams. This study examines the return airway of the 5107 fully mechanized top-coal caving (FMTC) face panel at the Yushupo Mine and analyzes roof coal stability under gob-side abutment pressure and mining-induced effects. A theoretical model based on limit equilibrium theory was established to reveal shear slip movement mechanisms in roof coal. A stability control technique for ultrathick seam FMTC roadways was proposed and validated through field monitoring.

## Engineering background

### Facial configuration and geological setting

**Fig. 2:**
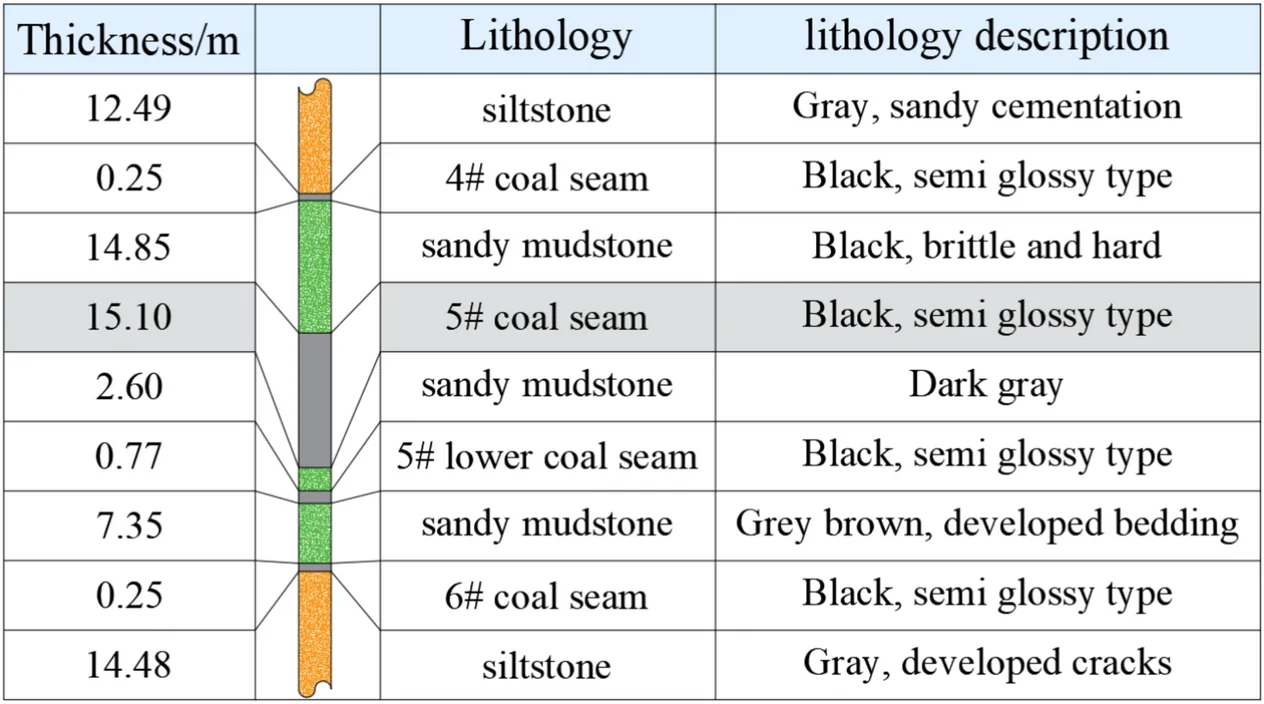
Comprehensive stratigraphic column.

The ZF5107 working face at the Yushupo Mine is located in the north-central section of Panel 1. With burial depths of 270–481 m and an average #5 coal seam thickness of 15.1 m, it represents a large-mining-height operation in ultrathick coal seams. While the coal seam has a relatively simple structure with strikes of 133–184° and dips of 5–15° (hardness coefficient f = 1.7), the roof coal contains five distinct parting layers, posing significant intraseam bedding separation risks. The immediate roof consists of 14.85 m of brittle-hard black sandy mudstone. The immediate floor comprises 2.6 m of gray‒black sandy mudstone underlain by 7.35 m of gray‒brown sandy mudstone featuring well-developed bedding planes and brittle behavior. The complete stratigraphic columns of the roof and floor strata are presented in Fig. 2.

### Roadway deformation characteristics

The ZF5107 return airway roadway was driven along the seam floor with a cross-section of 5.2 m (width) × 3.9 m (height). The roof support combines cable bolts, W-straps, and diamond-mesh screens. Five-m-long W-straps with six bolt holes were installed, with Φ21.8 × 6200 mm cable bolts spaced at 1000 mm intervals along each strap. Two supplementary Φ21.8 × 9200 mm cable bolts were installed 960 mm from the centreline between straps, with a distance of 1920 mm and an interrow spacing of 1920 × 1000 mm. The sidewall support employs rock bolts and a diamond mesh, with four Φ21.8 × 6200 mm bolts installed per side. The upper three bolts incorporate W-shaped bearing plates with 1000 × 1000 mm spacing.

**Fig. 3:**
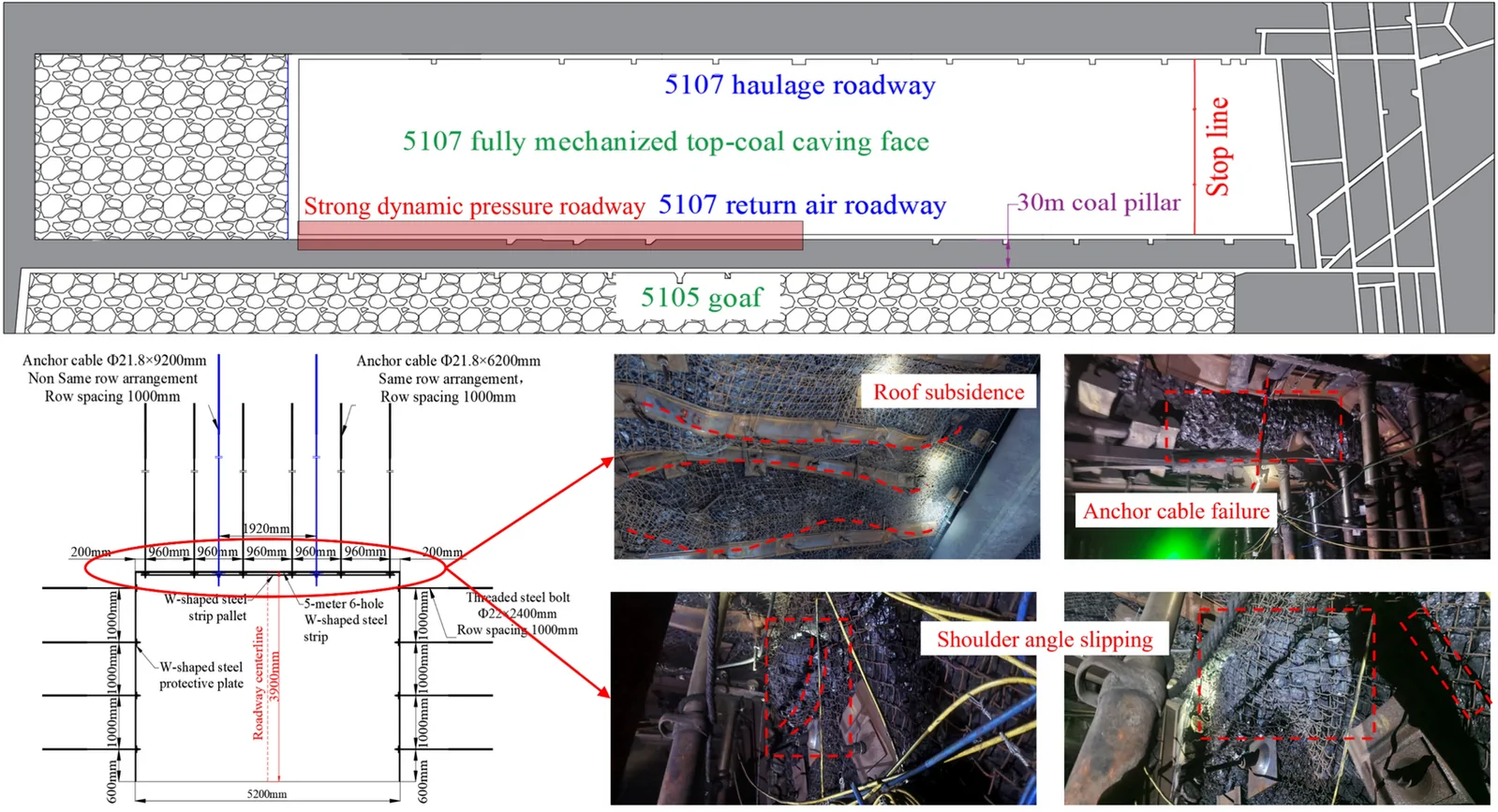
Roadway support configuration and critical surrounding rock failure characteristics.

Owing to the thick coal seam and intensive extraction at the FMTC face, the ZF5107 return airway roadway experiences severe ground pressure effects under the combined influence of adjacent gob areas and working face mining. This results in pronounced deformation and failure in advanced sections: localized fracture and collapse of the immediate roof create suspended mesh pockets, accompanied by support component degradation. The irregular failure patterns along the roadway axis included loose bearing plates, detached nuts, and exposed cable bolts/rock bolts. The floor exhibited fractured heave with central cracking, whereas both sidewall bases showed bulk uplift (Fig. 3).

**Fig. 4:**
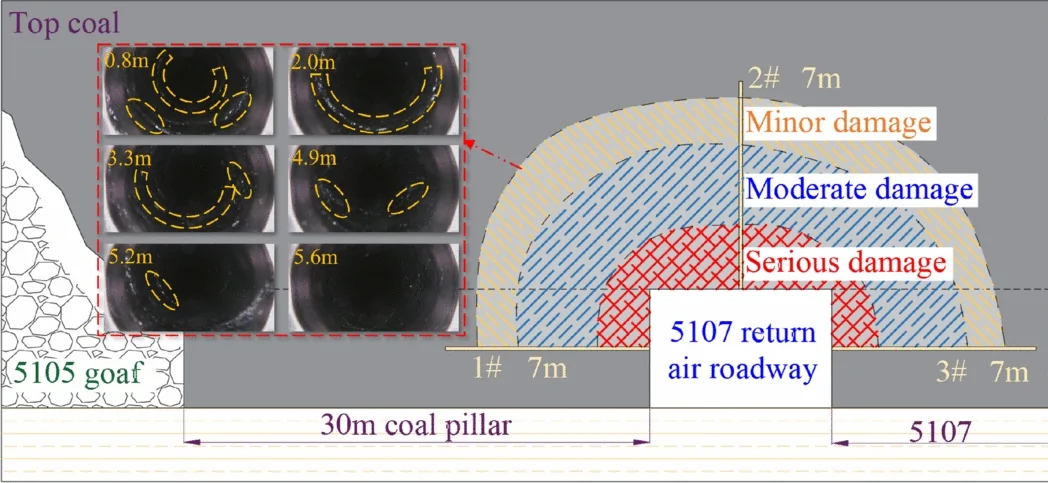
Surrounding rock observation results.

At a station 50 m ahead of the working face, fracture development was monitored via a YTJ-20 strata detector, revealing intense fracturing in roof coal under high-intensity mining abutment pressure, with intersecting macrofractures and dense microcracks. This produced distinct zones: a 0–2.0 m fractured zone, a 2.0–5.6 m plastic zone, and a stable zone beyond 5.6 m, as shown in Fig. 4.

During working face extraction, mining-induced stresses cause preexisting fractures and bedding planes in roof strata to propagate and coalesce into shear failure surfaces^22^. These surfaces dissect intact rock masses into unstable blocks^23^. Vertical stresses increase significantly within the roof, whereas horizontal stresses decrease due to excavation unloading. This deviatoric stress elevation drives unstable blocks toward the roadway free space. Concurrent roof subsidence and bedding separation subject bolts and cables to tension‒shear coupling, causing bending, fracture, or debonding from grout, ultimately compromising the roadway support system.

**Fig. 5:**
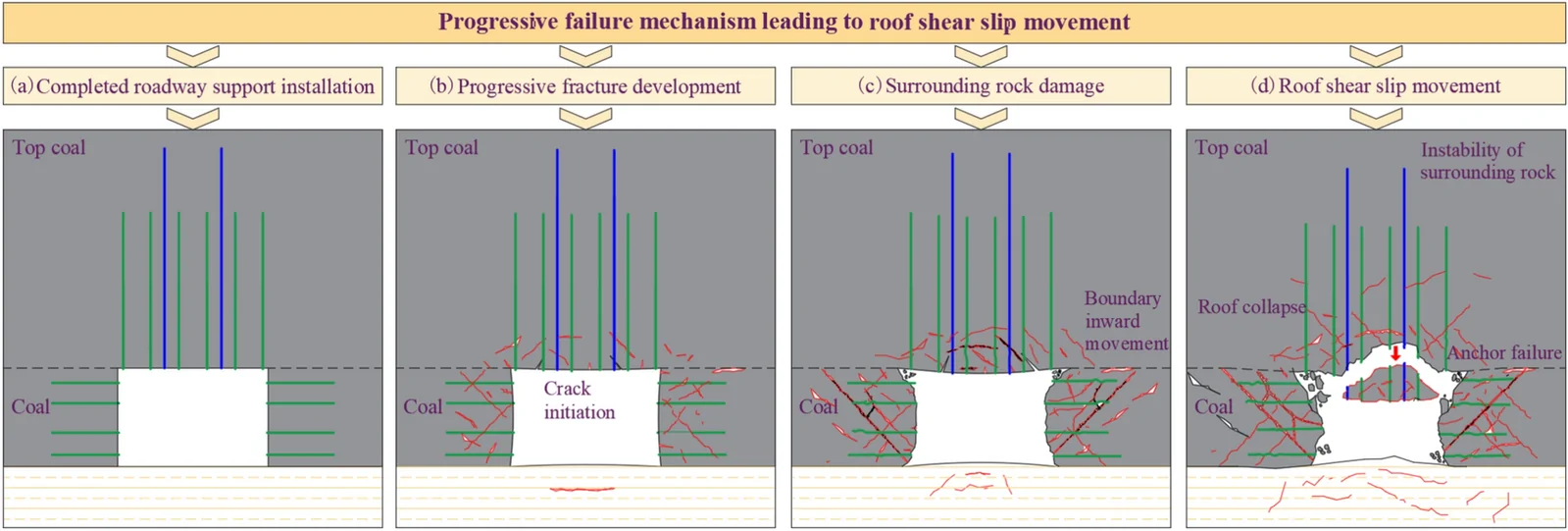
Progressive failure mechanism leading to roof shear slip movement.

At this point, roof displacement compresses floor strata, inducing floor heave that further destabilizes roof stress equilibrium, establishing synergistic roof–floor deformation. The movement of unstable blocks expands the fracture zone, with stress redistribution among adjacent blocks initiating multistage slip surface coalescence. This culminates in extensive roof shear slip movement and the precipitous deterioration of roadway stability, as shown in Fig. 5.

## Roof coal shear slip movement mechanism

### Failure criterion for roof shear slip

**Fig. 6:**
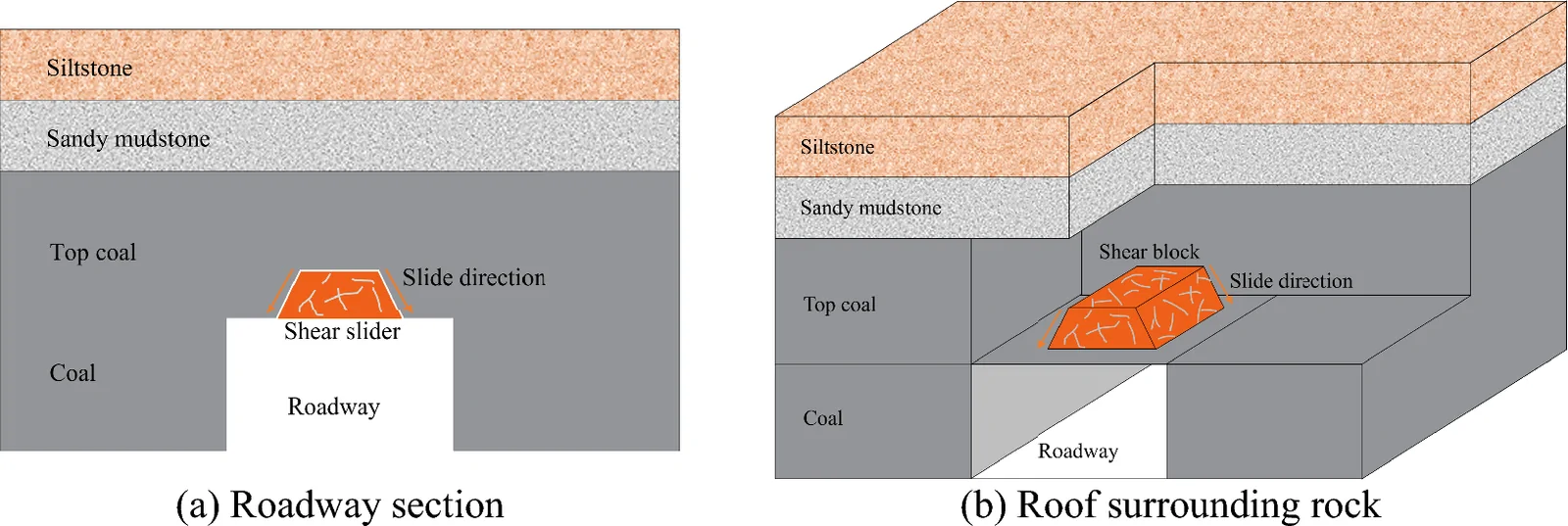
Schematic diagram of the roof shear slip movement.

Different failure modes exhibit distinct stress characteristics and progression mechanisms. Roof shear slip movement is caused primarily by shear stress exceeding shear strength^24,25^. Cracks initiate and propagate along the layer surface, penetrating the roadway surface and ultimately leading to roof shear slip movement^26^, as shown in Fig. 6.

Roof shear slip movement occurs when strata detach along weak structural planes (e.g., bedding, joints, or nascent fractures) under mining-induced stresses, gravity, and tectonic forces. Failure progresses through three stages: (a) Crack initiation: tensile fractures develop at the roof centerline due to bending stress. (b) Slip surface coalescence: Fractures propagate along bedding planes, forming continuous shear surfaces. (c) Block instability: Unstable blocks fail under self-weight and overburden pressure, manifesting as stratified sliding, cable bolt shear failure, and catastrophic collapse.

For roof shear slip movement, the theoretical shear strength *τ*_f_ based on the Mohr‒Coulomb criterion^21^ under 2D stress states is as follows:

1 $$\tau_{f} = c + \sigma_{n} \tan \varphi$$

where* c* is the cohesion of the shear plane, MPa; *σ*_n_ is the normal stress acting on the shear plane, MPa; and *φ* is the internal friction angle, °*.*

The safety factor *K* for roof shear slip movement is defined, which reflects the possibility of roof shear slip. Its value is equal to the ratio of the shear strength to the shear stress on the shear plane.

Failure criteria:

*K* < 1: Slip occurs along the shear plane;

*K* = 1: Critical equilibrium state;

*K* > 1: Stable condition.

2 $$K = \frac{\tau f}{\tau } = \frac{\int \mu d\sigma + \int c dl}{{\int d \tau }}$$

where *μ* is tan *φ*; *l* is the shear surface length, m; and *τ* is the shear stress acting on the shear surface, MPa.

Considering the strong mining dynamic disturbance, complex geological conditions and the discreteness of rock mass mechanical parameters in the engineering practice of ultra-thick coal seam mining, a certain safety margin is required in the support design to ensure the long-term stability of the roadway. Defining 1 ≤ K < 1.3 as a critical under stable state, with a high risk of instability under mining induced pressure disturbances, targeted reinforcement and support measures need to be taken.

**Fig. 7:**
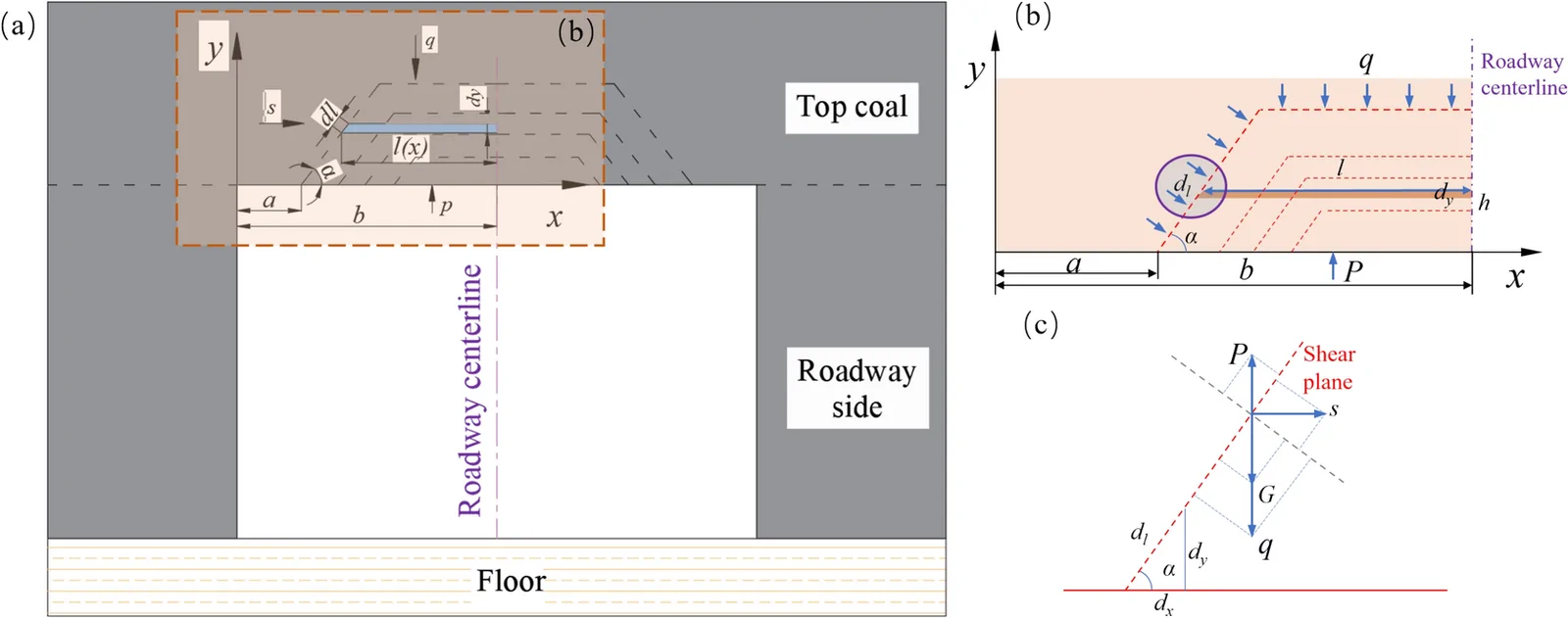
Roof shear slip movement model. (**a**) Shear slip block; (**b**) Force analysis; (**c**) Force analysis.

The roof shear slip movement in Fig. 6a is selected, limit equilibrium theory is applied, and a mechanical model of the roof shear slip block is established, as shown in Fig. 7 (Table 1).

**Table 1:** Model parameters.

Parameters	Definition
Vertical stress/*q*	Stress function on differential element
Horizontal stress/*s*	Stress function on differential element
Lateral pressure coefficient/*λ*	λ = Horizontal stress / Vertical stress
Element length/d_*l*_	Infinitesimal length of differential element
Element height/d_*y*_	Infinitesimal height of differential element
Shear plane function/y = (x-*a*)tanα	Geometric equation of failure surface
Support resistance/*P*	*P* = *p*·*l* (Total anchoring resistance)
Unit support resistance/*p*	Resistance per unit width
Slip line angle/*α*	Angle between slip line and horizontal
Shear initiation point/*a*	Horizontal distance from sidewall to shear origin
Half roadway width/*b*	Semiwidth of roadway
Diagonal length/*L*_1_	Length of shear block hypotenuse
Unit weight/*γ*	Bulk density of coal, kN/m^3^

Let the normal stress on the roof shear plane be *dσ* and the shear stress be dτ:

3 $$\begin{gathered} {\mathrm{d}}\sigma = \left( {q\cos \alpha + \gamma l\cos \alpha + s\sin \alpha - P\cos \alpha } \right){\mathrm{d}}y \hfill \\ {\mathrm{d}}\tau = \left( {q\sin \alpha + \gamma l\sin \alpha - s\cos \alpha - P\sin \alpha } \right){\mathrm{d}}y \hfill \\ \end{gathered}$$

Substituting Eq. (10) into the safety factor formula Eq. (9) yields the safety factor against roof shear slip movement as:

4 $$K = \frac{{\int {\mu \left( {q\cos \alpha + \gamma l\cos \alpha + s\sin \alpha - pl\cos \alpha } \right)dy} + \int {cdl} }}{{\int {\left( {q\sin \alpha + \gamma l\sin \alpha - s\cos \alpha - pl\sin \alpha } \right)dy} }}$$

here:

5 $$s = \lambda \cdot q,\;l = b - a - \frac{y}{\tan \alpha },\;\sin \alpha = \frac{dy}{{dl}},\;\cos \alpha = \frac{dx}{{dl}}$$

Substitution yields the following safety factor equation incorporating geometric parameters:

6 $$K = \frac{{\mu \int {\left[ {q + \left( {\gamma - p} \right)\left( {b - a - \frac{y}{\tan a}} \right)} \right]\frac{{x^{\prime}}}{{\sqrt {1 + x^{\prime 2} } }}dy} + \mu \int {s\frac{1}{{\sqrt {1 + x^{\prime 2} } }}dy} + c\int {\sqrt {1 + x^{\prime 2} } dy} }}{{\int {\left[ {q + \left( {\gamma - p} \right)\left( {b - a - \frac{y}{\tan a}} \right)} \right]\frac{1}{{\sqrt {1 + x^{\prime 2} } }}dy} - \int {s\frac{{y^{\prime}}}{{\sqrt {1 + x^{\prime 2} } }}dy} }}$$

**Fig. 8:**
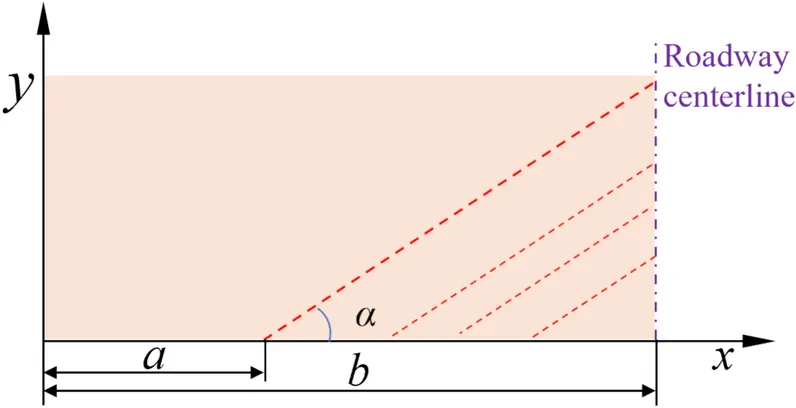
Simplified model of shear slip blocks.

The safety factor *K* against roof shear slip movement indicates whether shear failure planes will develop at a given roof location. Therefore, the shear slip block is simplified as a triangular geometry, as shown in Fig. 8, to facilitate solving the safety factor at arbitrary roof positions.

On the basis of geometric relationships:

7 $$\begin{aligned} \cos \alpha = & \frac{b - a}{{L_{1} }},\;\sin \alpha = \frac{y}{{L_{1} }} \\ L_{1}^{2} = & y^{2} + \left( {b - a} \right)^{2} \\ \end{aligned}$$

Equation (6) simplifies to:

8 $$K = \frac{{K_{1} }}{{K_{2} }}$$

Equation (8) can be solved separately to obtain:

9 $$\begin{aligned} K_{1} = & \mu \int\limits_{0}^{{y_{0} }} {\left[ {q + \left( {\gamma - p} \right)\left( {b - a - \frac{y}{{\tan \alpha }}} \right)} \right]} \frac{{x\prime }}{{\sqrt {1 + x^{{\prime 2}} } }}dy + \mu \int\limits_{0}^{{y_{0} }} {s\frac{1}{{\sqrt {1 + x^{{\prime 2}} } }}dy} + c\int\limits_{0}^{{y_{0} }} {\sqrt {1 + x\prime ^{2} } dy} \\ = & \mu y\left[ {q + \left( {\gamma - p} \right)\left( {b - a} \right)} \right]\cos \alpha - \frac{{\mu \left( {\gamma - p} \right)y^{2} \cos ^{2} \alpha }}{{2\sin \alpha }} + \mu \lambda qy\sin \alpha + \frac{{cy}}{{\sin \alpha }} \\ \end{aligned} $$$$

10 $$\begin{aligned} K_{2} = & \int\limits_{0}^{{y_{0} }} {\left[ {q + \left( {\gamma - p} \right)\left( {b - a - \frac{y}{{\tan \alpha }}} \right)} \right]} \frac{1}{{\sqrt {1 + x\prime ^{2} } }}dy - \int\limits_{0}^{{y_{0} }} {s\frac{{x^{\prime } }}{{\sqrt {1 + x^{{\prime 2}} } }}} dy \\ = & y\left[ {q + \left( {\gamma - p} \right)\left( {b - a} \right)} \right]\sin \alpha - \frac{{y^{2} \left( {\gamma - p} \right)\cos \alpha }}{2} - \lambda qy\cos \alpha \\ \end{aligned}$$

Substituting into Eq. (8) and simplifying the safety factor yields:

11 $$K_{{\mathrm{d}}} = \frac{{2\mu \lambda qy^{2} + \mu y\left( {b - a} \right)\left[ {2q + \left( {\gamma - p} \right)\left( {b - a} \right)} \right] + 2{\mathrm{c}}\left[ {y^{2} + \left( {b - a} \right)^{2} } \right]}}{{2qy^{2} + y^{2} \left( {\gamma - p} \right)\left( {b - a} \right) - 2\lambda qy\left( {b - a} \right)}}$$

Equation (18) incorporates fundamental parameters—roadway width, coal cohesion, friction angle, unit weight, roof load, and support resistance—which are readily obtainable. This facilitates the determination of the safety factor *K* against shear slip movement at any roof position.

**Fig. 9:**
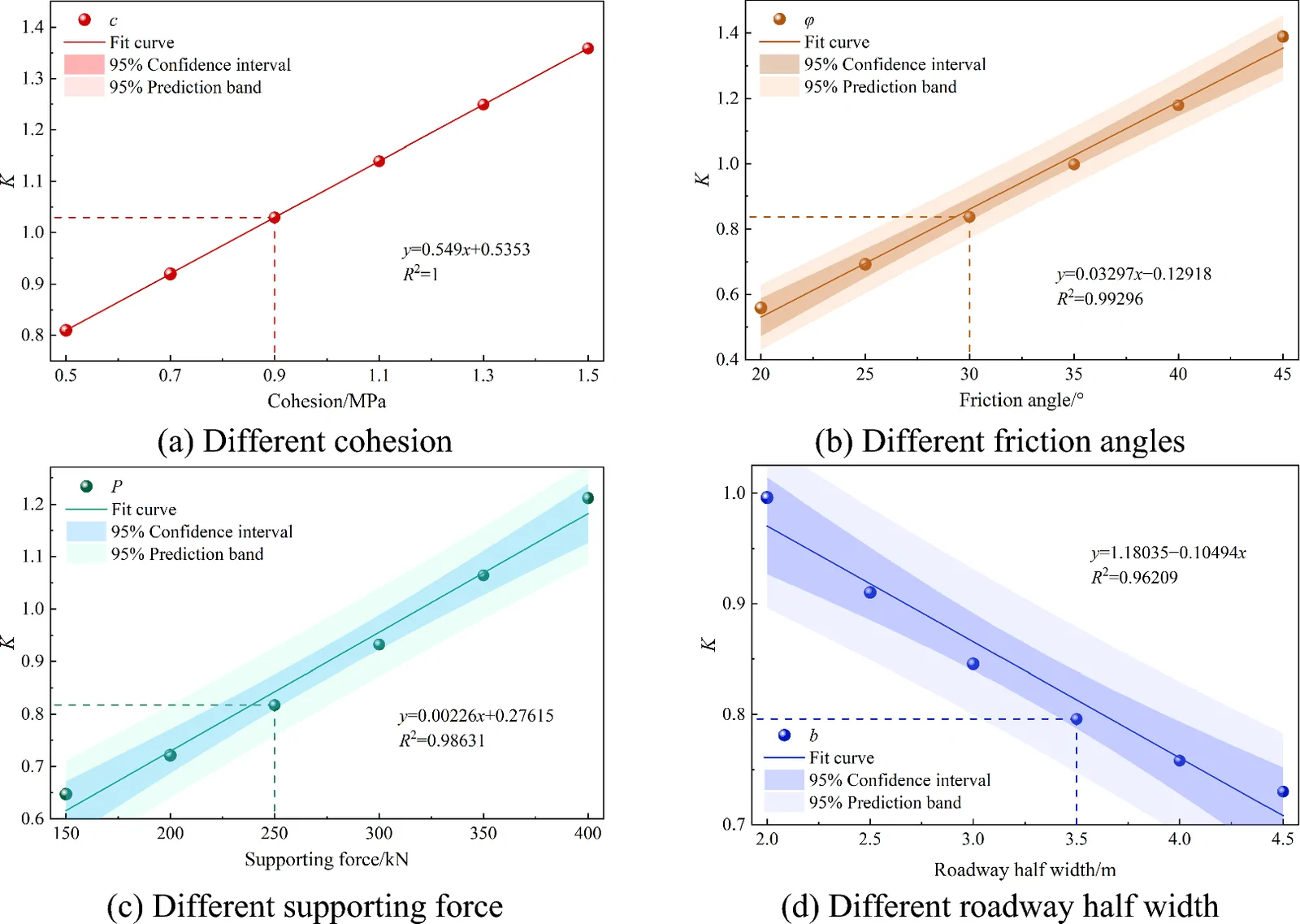
Influencing factors for the safety factor K.

Through parametric analysis by substituting the respective values, the influence patterns of various factors on the safety factor K can be determined, as shown in Fig. 9.

With respect to the intrinsic properties of coal, the safety factor against roof shear slip movement increases with both cohesion and friction angle, with cohesion having a more significant influence. The safety factor linearly increases with increasing cohesion. Under external conditions, the safety factor increases proportionally with the bolt-cable support pressure. Conversely, it progressively decreases with increasing roadway width.

It should be noted that the proposed limit equilibrium model incorporates several key assumptions: (1) the roof is a homogeneous isotropic elastoplastic medium conforming to the Mohr–Coulomb criterion; (2) the shear slip surface is simplified as a plane; (3) the roof stress state is simplified as a plane strain problem, matching the roadway’s geometric characteristics; (4) the bolt-cable support effect is equivalent to uniformly distributed support resistance calculated from actual support parameters. Under strong mining dynamic pressure, this model applies to shear slip stability analysis of coal-seam roof roadways in ultra-thick coal seam caving faces, for cases involving severe dynamic impact loads (e.g., rockbursts), further dynamic continuous-discontinuous modeling is required.

### Roof shear slip movement safety factor distribution

To quantify the safety factor analytically, the vertical stress in roof coal requires detailed examination. Treating this as a plane strain problem^27^. The stress distribution around the roadway follows elastic theory^28^, which provides the stress solution around an opening under biaxial anisotropic stress:

12 $$\left\{ \begin{gathered} \sigma_{r} = \frac{\gamma H}{2}(1 + \lambda )\left( {1 - \frac{{r_{1}^{2} }}{{r^{2} }}} \right) - \frac{\gamma H}{2}(1 - \lambda )\left( {1 - 4\frac{{r_{1}^{2} }}{{r^{2} }} + 3\frac{{r_{1}^{4} }}{{r^{4} }}} \right)\cos 2\theta \hfill \\ \sigma_{\theta } = \frac{\gamma H}{2}(1 + \lambda )\left( {1 + \frac{{r_{1}^{2} }}{{r^{2} }}} \right) + \frac{\gamma H}{2}(1 - \lambda )\left( {1 + 3\frac{{r_{1}^{4} }}{{r^{4} }}} \right)\cos 2\theta \hfill \\ \end{gathered} \right.$$

where *σ*_*θ*_ is the tangential stress; *σ*_*r*_ is the radial stress; and *r*_1_ is the equivalent roadway radius.

where the lateral pressure coefficient *λ* is determined from the Hoek‒Brown regression curve envelope^29^:

13 $$\lambda = \frac{100}{H} + 0.3$$

Then, at *θ* = 90°, the vertical stress on roof coal (q) is:

14 $$\sigma = \frac{\gamma H}{2}\left[ {(1 + \lambda )\left( {1 - \frac{{r_{1}^{2} }}{{r^{2} }}} \right) - (1 - \lambda )\left( {1 - 4\frac{{r_{1}^{2} }}{{r^{2} }} + 3\frac{{r_{1}^{4} }}{{r^{4} }}} \right)} \right]$$

The occurrence characteristics of the roof are summarized in Table 2. Among them, the cohesion and internal friction angle of coal and rock specimens, as well as the interlayer friction coefficient, were determined through systematic laboratory tests. The support resistance and lateral pressure coefficient were obtained by combining field in-situ measurements with theoretical calculation methods.

**Table 2:** Roof occurrence characteristics.

Parameters	Value
Cohesion/*c,* MPa	1.63
Internal friction angle/*φ, °*	42.60
Average unit weight of overburden/γ, kN/m^3^	26
Roadway dimensions/m	5.2 × 3.9
Roadway burial depth/*H, m*	428
Interlayer friction coefficient/*f*	1.70
Roof tensile strength/*σ*_Rt_, MPa	1.06

**Fig. 10:**
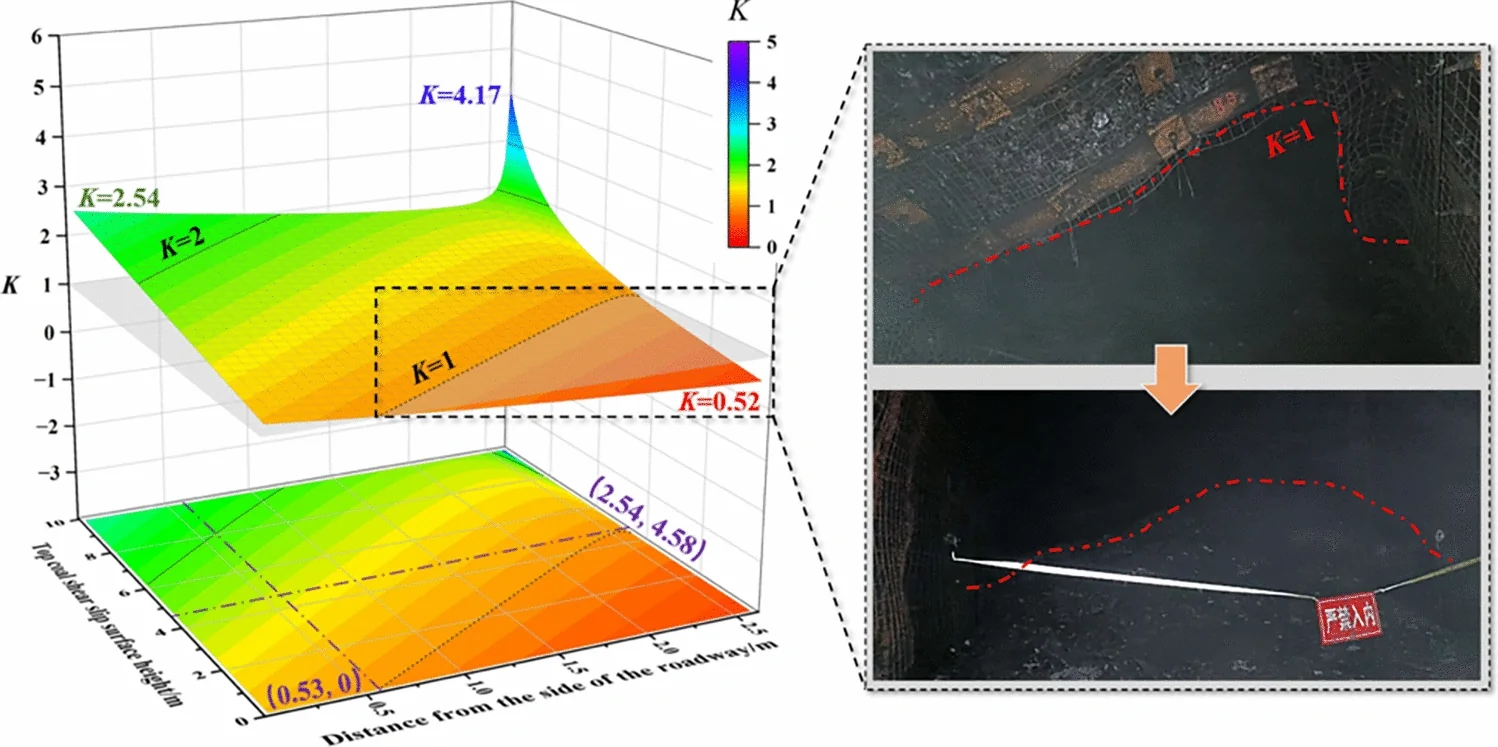
Distribution of safety factors against.

By incorporating the roadway width, coal cohesion, friction angle, unit weight, roof load, and support resistance parameters along with Eqs. (13)–(14) into Eq. (11), the safety factor distribution against shear slip movement was determined as a function of the distance from the sidewalls and roof coal thickness, as shown in Fig. 10. The distribution of the safety factor for top coal shearing and caving follows the following pattern:

1. At a constant shear slip failure plane height, the safety factors increase progressively from the roof centerline toward the sidewalls. Similarly, at fixed lateral positions, factors generally increase with increasing height.
2. The maximum slip height reaches 4.58 m at 0.06 m from the centerline,which is within the field-measured plastic zone range of 2.0–5.6 m, and the boundary of the theoretical shear slip zone is highly consistent with the boundary between the plastic zone and the stable zone measured on site. Severe roof subsidence occurs because of the combined overburden pressure, self-weight, and elevated lateral stresses from gob areas.
3. Shear failure initiates 0.53 m from the sidewalls, with decreasing safety factors toward the centerline, indicating greater instability mid-roof, which is consistent with the field phenomenon that the most severe roof subsidence and collapse occur in the middle of the roof.
4. Safety factors increase with increasing slip plane height, indicating greater stability. This confirms that higher anchoring elevations provide enhanced safety buffers. The optimal support should extend beyond potential slip planes, with additional height margins further improving security.

## Influencing factors of roof coal stability

**Fig. 11:**
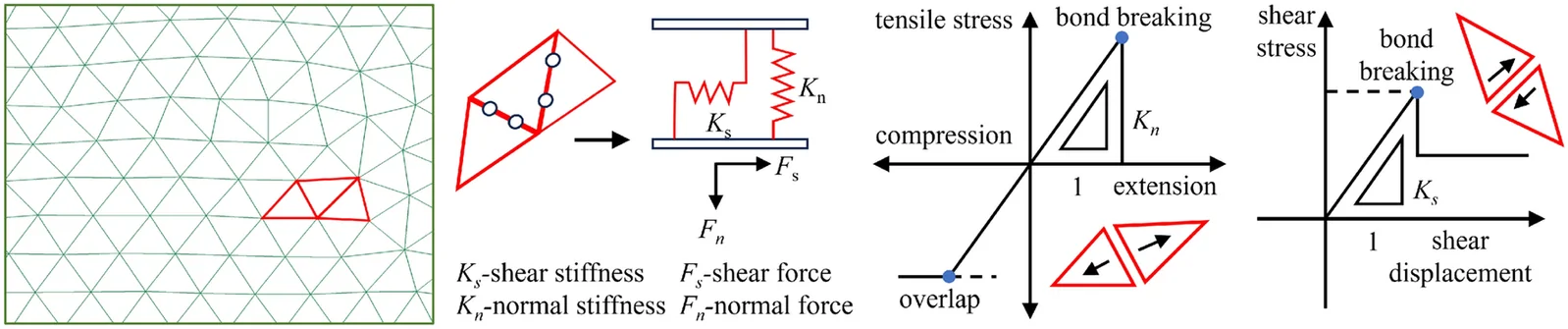
UDEC modeling of fracture development in strata.

Based on the theoretical analysis of the instability mechanism of roof shear slip in the previous section, this section uses the Discrete Element Method (DEM) to conduct numerical simulation research, systematically analyzing the influence of roof coal cohesion, gangue layer characteristics, and support schemes on the evolution of roof shear slip. The DEM effectively accounts for inherent rock fractures, making it advantageous for simulating large deformations—such as roof shear slip movement and floor heave—induced by mining disturbances^30,31^. The DEM accurately captures geometric features, with fracture patterns and constitutive behaviors between blocks illustrated in Fig. 11. Tensile failure occurs when the normal tensile stress exceeds the joint tensile strength *t*, resulting in *σ*_n_ = 0. The shear stress *τ*_s_ is governed by the cohesion *c* and friction angle *ϕ* between the contacting blocks. Interface deformation is determined by the contact stiffnesses *K*_n_ (normal) and *K*_*s*_ (shear). When the shear stress acting on the joint exceeds the maximum shear strength, the joint will undergo shear slip failure.

**Fig. 12:**
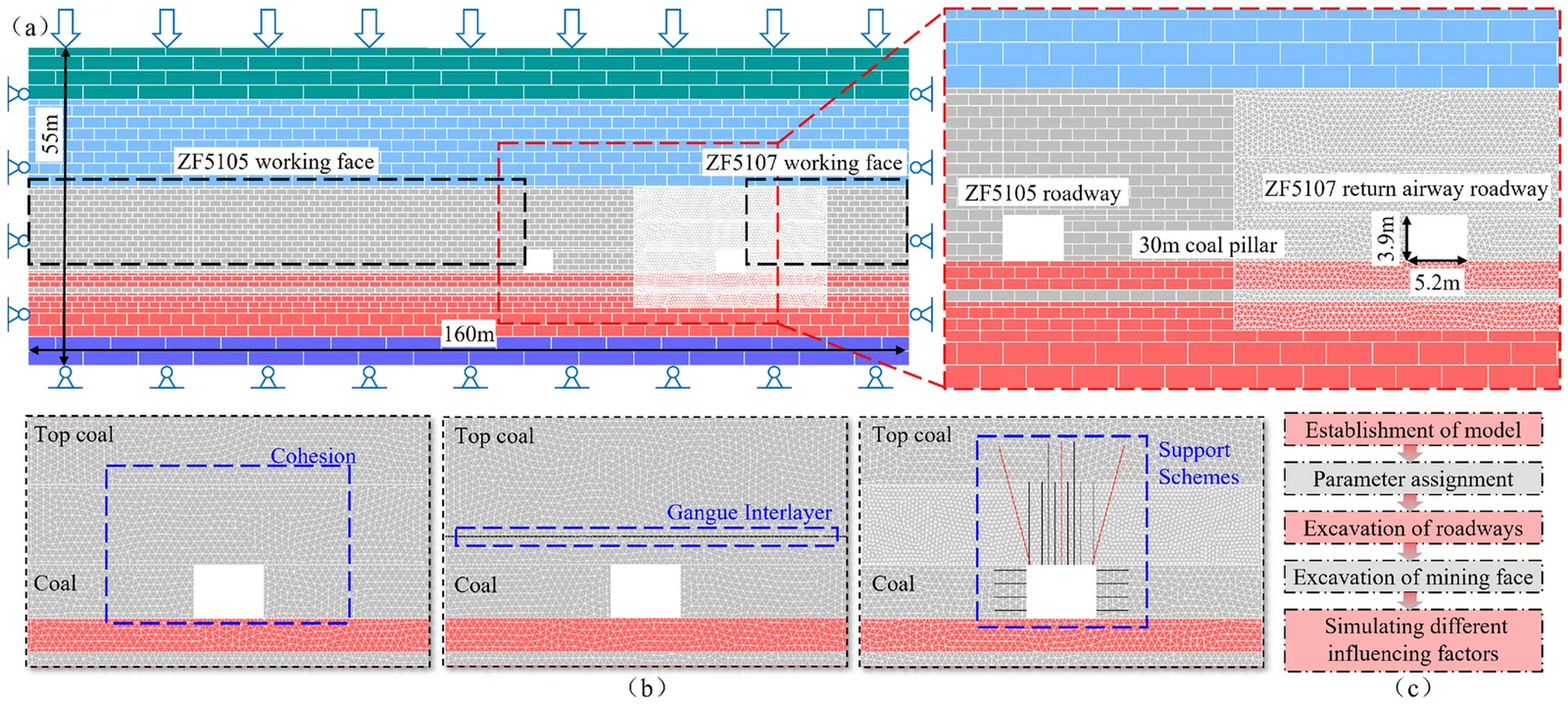
Numerical calculation model. (**a**) Numerical model and local enlarged view; (**b**) Numerical simulation scheme; (**c**) Calculation sequence.

On the basis of the geological and operational conditions of the ZF5107 working face, a numerical model was established via UDEC software^32^ as show in Fig. 12, the mechanical parameters of the numerical model are determined through laboratory tests, field in-situ measurements. This model simulates the influence of cohesion, the gangue interlayer and roadway parameters on shear block movement in roof coal. The excavation sequence replicates field practice. First, the ZF5105 and ZF5107 return airway roadways are excavated simultaneously. The ZF5105 working face was excavated. To simulate the postpeak softening behavior and shear dilation characteristics of roadway surrounding rock and joints, a strain-softening constitutive model was applied to coal seams and roof/floor strata. The numerical simulation parameters are listed in Table 3. The mechanical parameters of the numerical model are determined through laboratory tests, field in-situ measurements.

**Table 3:** Numerical simulation parameters.

Model Parameters	Value
Dimensions/m	160 × 55
Boundary constraints	Fixed
Bottom constraint	Fixed
Top constraint	Free
Upper boundary stress/MPa	9.82

### Influence of coal cohesion

**Fig. 13:**
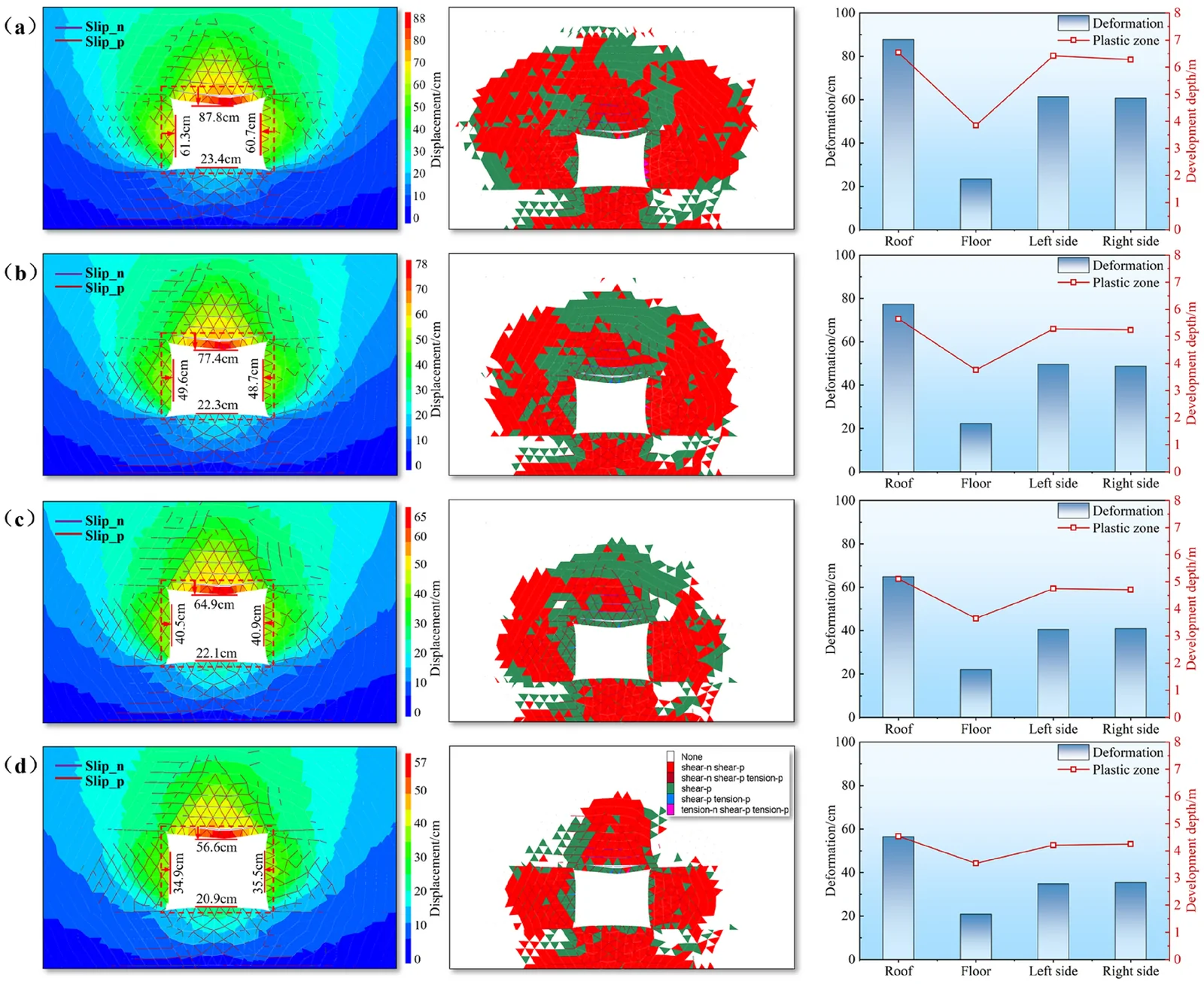
Roadway displacement characteristics under varying cohesion conditions. (**a**) 1.25 MPa; (**b**) 1.50 MPa; (**c**) 1.75 MPa; (**d**) 2.00 MPa.

In accordance with the Mohr‒Coulomb criterion, the shear strength increases with cohesion^33^. Higher cohesion enhances sidewall shear resistance, increasing safety factors against sliding. When cohesion increased from 1.25 to 2.0 MPa, the roadway displacement characteristics changed (Fig. 13).

Displacement at the sidewalls and roof-floor midpoints reveals the following: (1) Surrounding rock deformation decreases with increasing cohesion; (2) At 1.25 MPa cohesion, roof subsidence of 87.8 cm and sidewall convergence of 123.0 cm. At 2.00 MPa, subsidence was 56.6 cm, and convergence was 70.4 cm. Increasing cohesion from 1.25 to 2.00 MPa reduced roof-floor closure by 35.5% and sidewall convergence by 42.8%. When other roadway conditions remain unchanged, greater cohesion reduces surrounding rock deformation and enhances stability under constant conditions. (3) When the cohesion is 1.75 MPa, there is a turning point in the decrease in displacement amplitude. From 1.25–1.75 MPa, the displacement of the roadway sidewall decreases significantly; from 1.75–2.00 MPa, the reduction rate gradually attenuates. (4) Enhanced cohesion directly improves the shear resistance of coal, effectively preventing sliding block failure. This principle provides a theoretical basis for grouting modification of the surrounding rock.

In summary, the greater the cohesion is, the stronger the shear strength of the surrounding rock itself. From the perspective of onsite prevention and control, it is possible to improve the mechanical properties of the surrounding rock and enhance the stability of the roadway by reasonably using grouting reinforcement technology.

### Influence of gangue interlayer

**Fig. 14:**
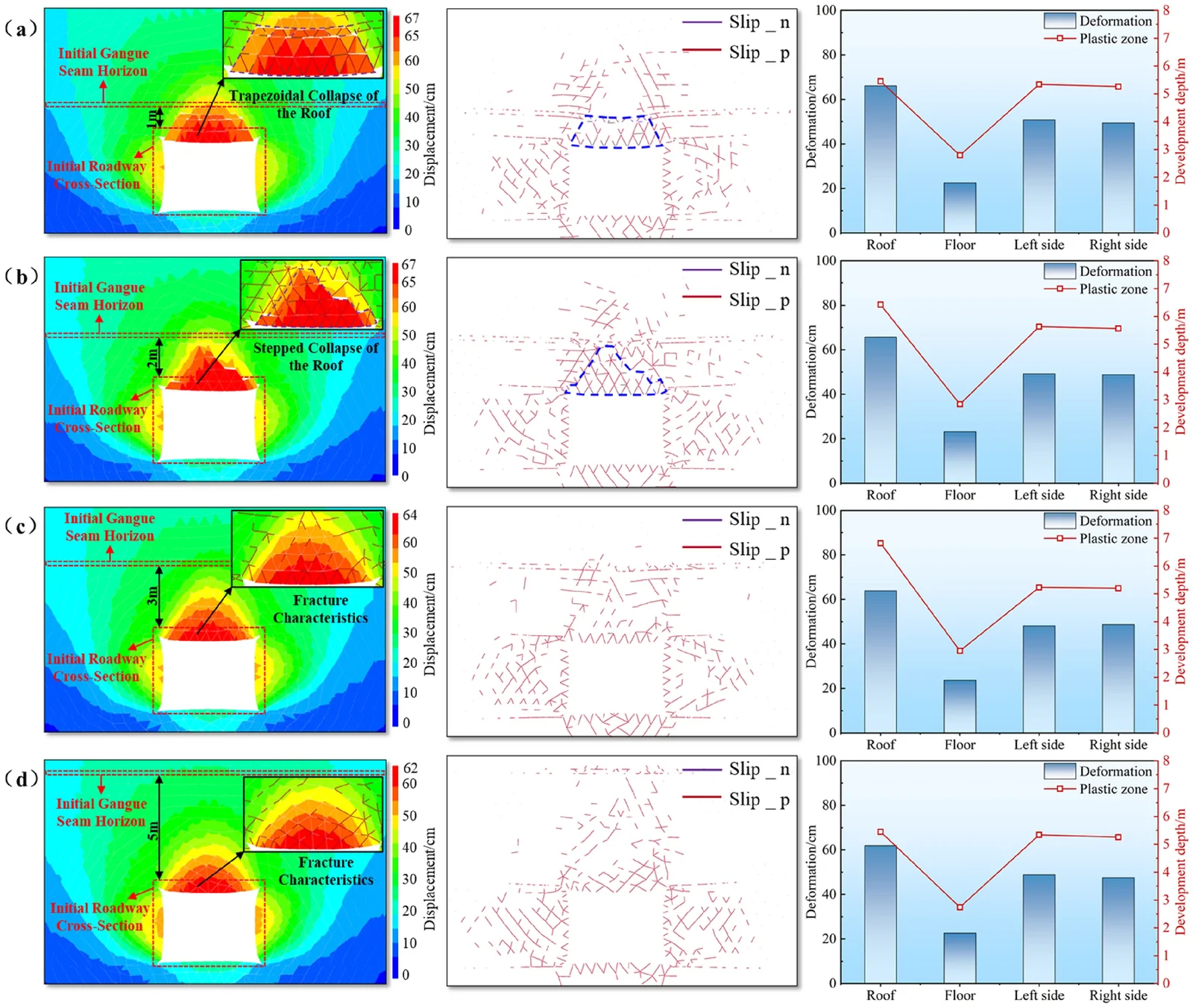
Influence of the gangue interlayer on roof coal. (**a**) 1 m; (**b**) 2 m; (**c**) 3 m; (**d**) 5 m.

Five gangue interlayer layers in the ZF5107 working face roof coal significantly altered the stress distribution, failure modes, and shear behavior. Parting-coal interfaces become preferential slip paths, dividing roof coal into segments and affecting shear slip block dimensions^34^. With the gangue interlayer positioned at 1 m, 2 m, 3 m, and 5 m within the roof coal, the postexcavation stability characteristics are shown in Fig. 14. The gangue interlayer in the roof coal is sandy mudstone, and its mechanical property parameters are obtained from laboratory tests of standard core specimens collected on site.

Figure 14 shows the following: (1) When the gangue interlayer is located 1 m above the roof coal, partings near the roof experience high deviatoric stresses and undergo tensile‒shear failure. This creates weak zones triggering rapid sliding of the underlying coal, resulting in trapezoidal subsidence. (2) At a height of 2 m, partings divide the roof coal: the upper section gains support, whereas the lower section develops stepped slip surfaces, the presence of interlayers causes layered movement of shear blocks, significantly increasing the height and extent of roof shear slip movement. (3) At heights of 3 and 5 m, their relatively higher strength than that of the roof coal allows the upper interlayered rock to bear part of the overburden load. However, if the interlayered rock suddenly fractures, it may cause a sudden increase in stress on the lower coal, triggering sudden shear failure of the roof coal.

In summary, the presence of gangue interlayers affects the shear slip movement characteristics of the roof to some extent. First, the weak coal-gangue interface coefficient acts as a preferential path for shear slip surface propagation, significantly lowering the overall shear strength of the roof. Second, the mismatch in elastic modulus between the gangue interlayer and coal mass induces stress concentration at the interface, which accelerates the initiation and coalescence of shear cracks. Third, the interlayer divides the roof coal into independent layered cantilever structures, which aggravates bending deformation and interlayer dislocation, further expanding the scale of shear slip and affecting the overall stability of the roof coal.

### Influence of support schemes

**Fig. 15:**
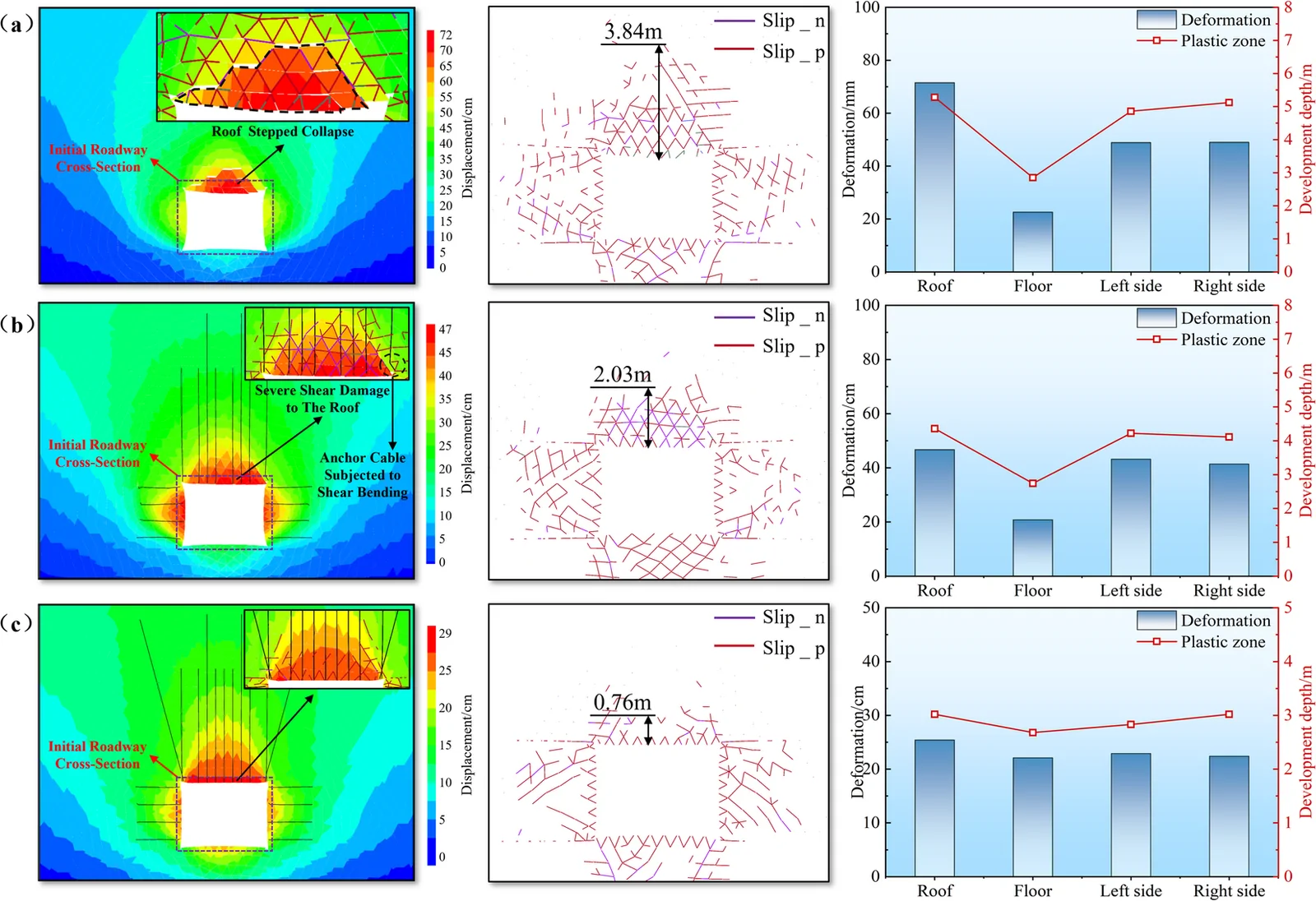
Influence of support schemes on roof coal. (**a**) No support; (**b**) Original support; (**c**) Tilted anchor cable support.

To evaluate support reliability under excavation and mining stresses, numerical simulations were used to analyze shear slip movement behavior under three schemes: unsupported, conventional bolt-cable support, and roof-inclined fully grouted anchor cables (Fig. 15).

Figure 15 revealed the following: (1) Unsupported conditions: roof coal bears loads without external restraints. Fractures develop to 3.84 m in height, with preexisting cracks rapidly coalescing into continuous slip surfaces, causing a stepped slip of fragmented coal. (2) Conventional support: Anchoring deep stable strata provides limited enhancement. Although long cables crossing potential slip planes improve shear resistance, cable bending under shear forces causes significant displacement, which compromises anchorage. Fractures develop to 2.03 m with 46.7 cm of subsidence. (3) Inclined grouted cables: Cables directly traverse shear planes to resist shear stresses. Full-length grout injection fills coal fractures, enhancing cohesion and shear strength. The fracture height decreases to 0.76 m, with only 25.4 cm of subsidence—a 43.47% improvement over conventional support. This finding shows that the addition of inclined fully grouted anchor cables can effectively control the deformation and destruction of the surrounding rock in thick coal seam roadways, offering valuable guidance for similar conditions.

## Coordinated control technology for roadway surrounding rock in thick coal seams

**Fig. 16:**
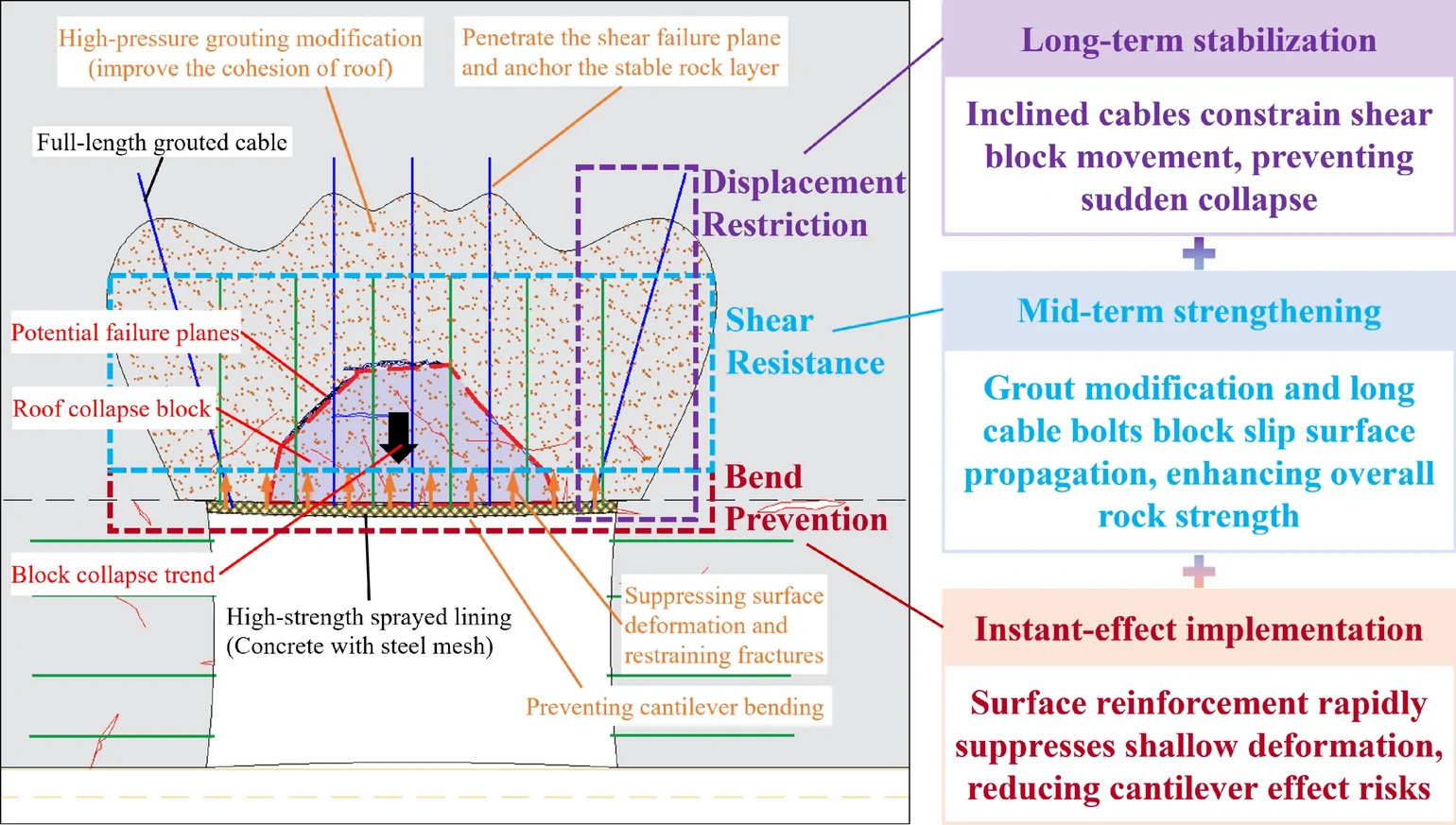
Cooperative control strategy.

In ultrathick coal seams, low-strength roof coal undergoes stress redistribution during mining, altering the principal stress orientation. This induces tensile‒shear composite fractures that propagate along bedding planes, weakening the load-bearing capacity. The main manifestation is the shear slip and destruction of stepped/wedge-shaped blocks^33,35^. Therefore, we propose a coordinated “bend prevention–shear resistance–displacement restriction” strategy, as shown in Fig. 16: (1) Bend prevention: surface reinforcement rapidly suppresses shallow deformation, reducing cantilever effect risks; (2) shear resistance: grout modification and long cable bolts block slip surface propagation, enhancing overall rock strength; and (3) displacement restriction: inclined cables constrain shear block movement, preventing sudden collapse and ensuring long-term stability. Through three-stage, multilevel prevention and control technology, comprehensive control of roof coal seam shear block caving in extrathick coal seams can be achieved.

### Roadway stability control scheme

1. “Bend prevention-shear resistance-displacement restriction” cooperative control strategy

Bend Prevention (Surface Reinforcement): Sprayed concrete with steel mesh forms a high-strength spray layer lining that suppresses the initiation and propagation of tensile cracks, preventing bending deformation.

Shear resistance (deep modification): High-pressure grouting fills bedding fractures and cements loose rock, enhancing cohesion^36^. Long cable bolts penetrating potential failure planes anchor into stable strata, creating a “shear-resistant skeleton”^37^, which improves deep rock shear capacity and reduces shear block volume.

Displacement Restriction (Local Reinforcement): Inclined cables (20° ~ 30°) installed at roof shoulders form spatial trusses that constrain residual deformation and block sliding movement. Yieldable supports dissipate deformation energy, preventing stress concentration-induced shear slip movement.

(2) Design scheme for full-length grouted cable reinforcement support

**Fig. 17:**
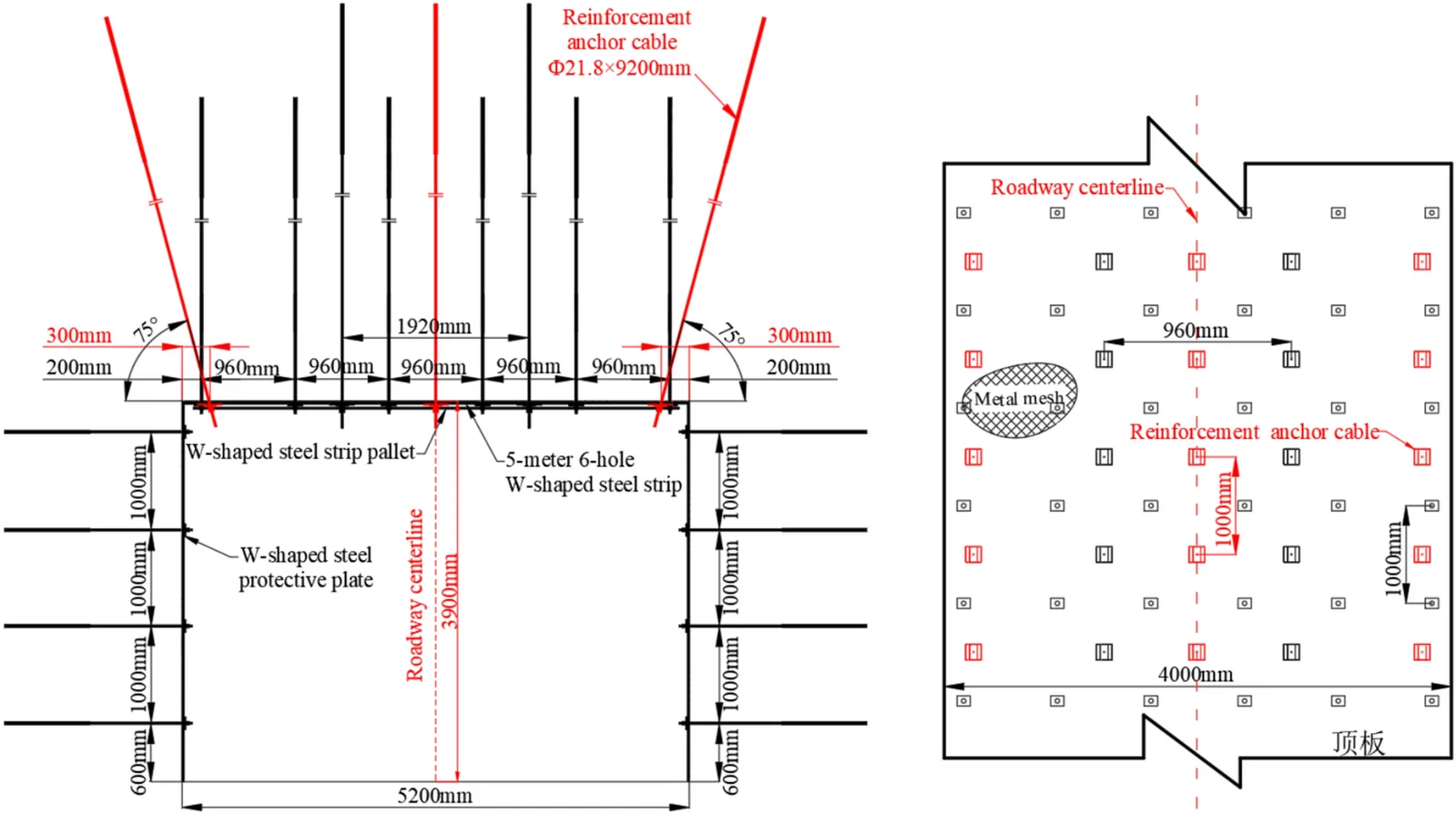
Reinforcement support cross section.

On the basis of the mechanism of shear slip movement of the roadway roof in the dynamic pressure zone of the thick coal seam and the technical scheme for controlling the stability of the surrounding rock, combined with the actual geological conditions of the Yushu Slope Coal Industry, the ZF5107 return airway employs a “6 + 2 + 3 full-length grouted cable” pattern, 6 + 2 is the original support scheme for the roof, and on this basis, 3 full-length grouting anchor cables are added and the outermost cables inclined at 15° as show in Fig. 17.

A full-length grouted cable bolt, measuring Ф 21.8 × 9200 mm, is installed centrally within the top coal section. Additionally, one full-length grouted cable bolt is installed at each side of the roadway, positioned 300 mm outward from the ribs at a 15° inclination angle, totaling three supplementary cable bolts. The reinforcement spacing is 1000 mm. The cable bolt ends are secured via resin cartridges, while the free lengths are fully grouted.

The primary constituents and key parameters of the cement grout used in the full-length grouting process for the cable bolts are presented in Table 4.

**Table 4:** Parameters of the grouting scheme.

Parameters	Requirement
Water-cement ratio	1:2
Additive dosage	ACZ-1 type(8% by mass)
Grouting pressure/MPa	5 ~ 8
Grouting duration/min	15–20
Pumping distance/mm	≥ 2000
Maximum reaction temperature/°C	≤ 38

**Fig. 18:**
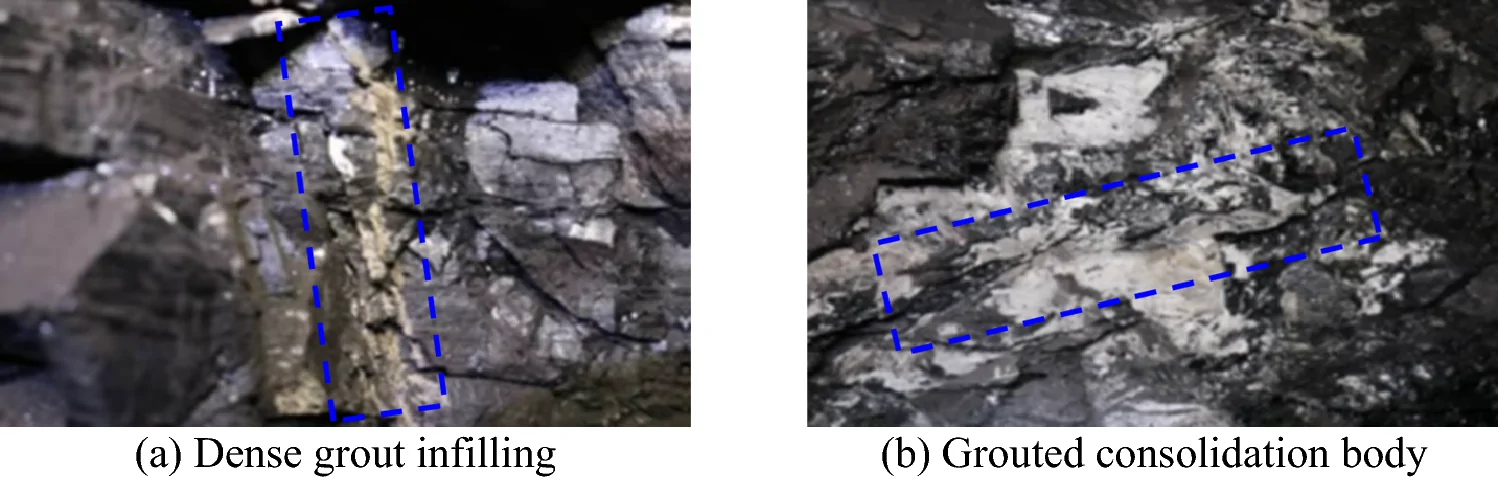
Grouting consolidation effects.

The cement slurry has high injectability, strong bonding, low porosity, and high impermeability (final permeability coefficient of 10^−8^–10^−10^ cm/s). Postgrouting, inherent fractures are effectively filled, significantly enhancing integrity and stability, as shown in Fig. 18.

### Field trial monitoring and evaluation

**Fig. 19:**
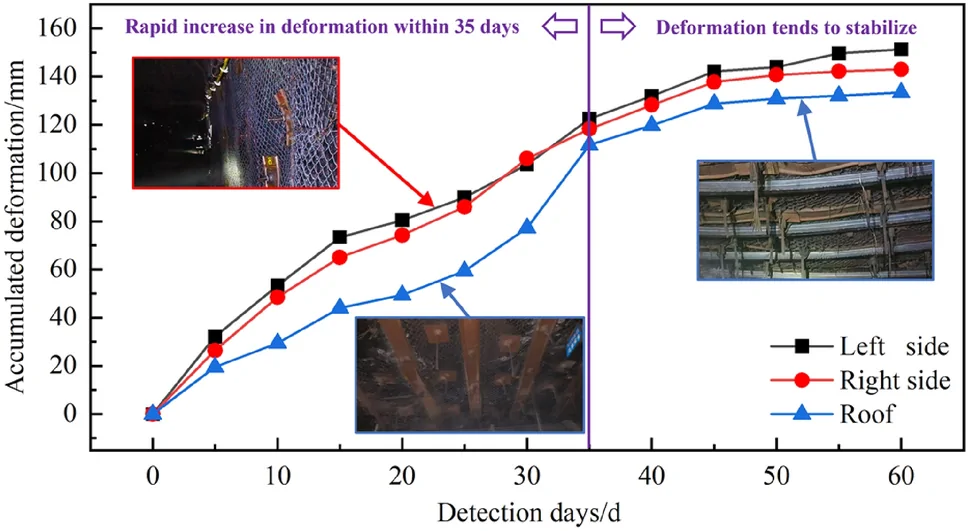
Roadway deformation monitoring curves.

To validate the feasibility and rationality of the synergistic “bend prevention–shear resistance–displacement restriction” control technology and the full-length grouted cable bolt reinforcement support scheme, industrial trials were conducted at the tailgate of the ZF5107 longwall panel. The monitored roof separation, roof-to-floor convergence, and roadway sidewall convergence within the 0–100 m zone ahead of the face over a 60-day period are presented in Fig. 19.

**Fig. 20:**
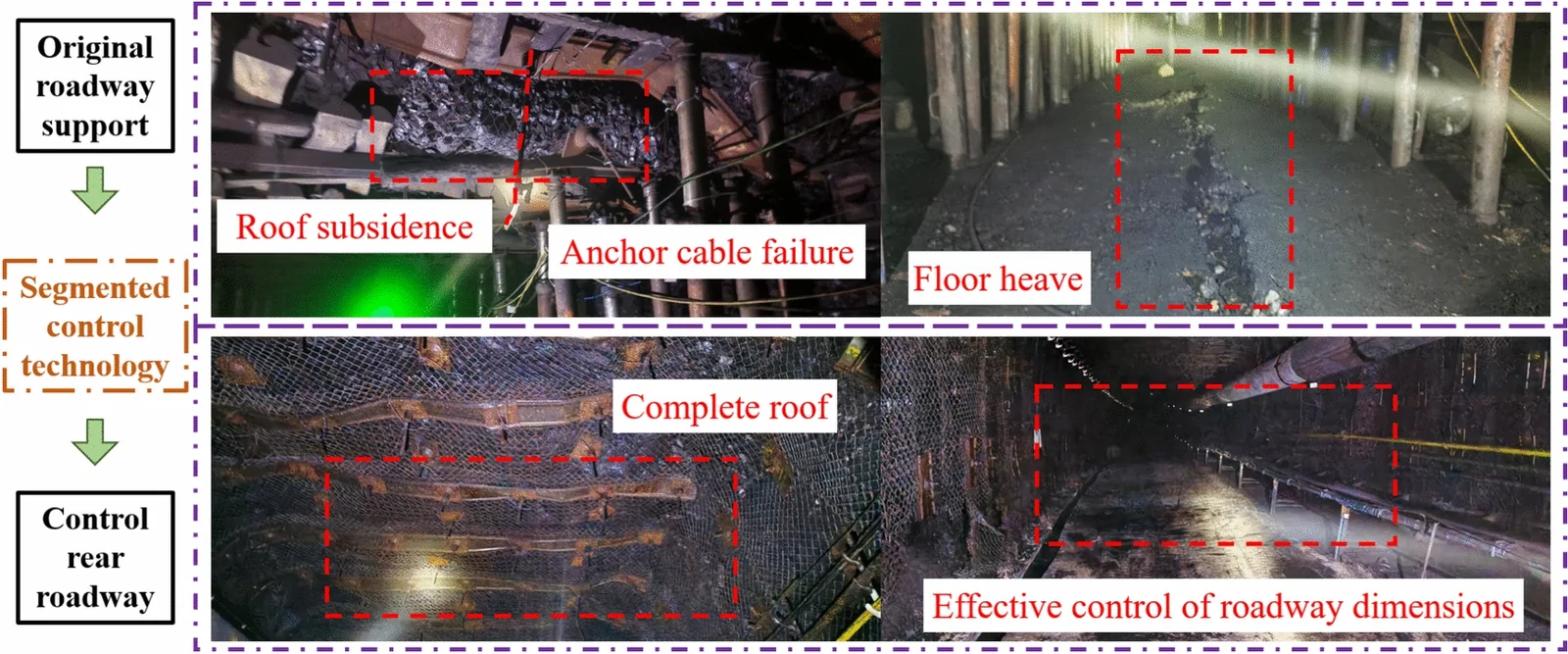
Control effect of roadway.

To further substantiate the effectiveness of the proposed control technology, statistical comparative data were analyzed. Under the original support scheme, the roadway experienced severe deformation within 30 days of excavation, with maximum roof subsidence exceeding 297 mm and sidewall convergence reaching up to 653 mm, often accompanied by anchor cable shear failure. Following the implementation of the synergistic control technology, roadway convergence increased rapidly within the first 35 days. The maximum recorded roof subsidence convergence was drastically reduced to 133.5 mm, and roadway sidewall convergence dropped to 151.4 mm and 143.0 mm (left and right), respectively. This represents an approximate 55% reduction in overall surrounding rock deformation. Moreover, the deformation rate stabilized significantly after 35 days (Fig. 20). The technology enabled effective control of roadway convergence within the high-stress zone of the ultrathick coal seam, thus validating the rationality of the control approach.

## Main conclusions

1. Roof shear slip movement is a phenomenon where the roof strata undergo large-scale caving failure along weak structural planes under mining-induced stresses. Under the combined influence of mining-induced stress, gravitational stress, and tectonic stress, preexisting fractures and bedding planes progressively propagate and coalesce, forming through-going shear failure surfaces that dissect the coal mass into unstable blocks, ultimately leading to roof shear slip movement.
2. A theoretical mechanical model for shear failure blocks in roadway roof coal was established via an exact solution method on the basis of limit equilibrium analysis. This model quantifies the distribution pattern of the safety factor for the roof coal shear block: the shear failure plane initiates at 0.53 m from the roadway sidewall. The safety factor is minimal near the roadway centerline, indicating that the highest susceptibility to roof shear slip movement occurs in the middle of the roadway section. The maximum height of the shear failure zone is 4.37 m; consequently, the lowest point of the cable bolt anchorage zone must extend beyond this critical height.
3. Through theoretical analysis and numerical simulation, the key controlling factors of roof stability are clarified: the cohesion of roof coal is the core intrinsic factor affecting shear strength; the gangue interlayer is the preferential propagation path of shear failure surfaces, and its position determines the development scale of the slip zone; the inclined full-length grouted cable can directly cross the potential shear slip surface to resist shear stress, and the grouting modification can enhance the cohesion of coal mass, which is the most effective control method for roof shear slip instability.
4. A synergistic “bend prevention–shear resistance–displacement restriction” control technology is proposed, and a targeted full-length grouted cable reinforcement scheme is developed. Field application shows that the technology reduces the overall surrounding rock deformation by more than 55%, and effectively controls the large deformation of the roadway under strong dynamic pressure. This technology provides a practical engineering solution for the stability control of roadways in ultra-thick coal seam fully mechanized caving mining, and has important reference value and popularization significance for similar geological and mining conditions.

## Data Availability

Data will be made available on request.
